# Multicenter study on CT-based Radiomics for predicting severity and delayed recovery in *Mycoplasma pneumoniae* pneumonia

**DOI:** 10.3389/fmed.2025.1652653

**Published:** 2025-11-05

**Authors:** Qian Li, Zi-Jun Song, Wenjing Chen, Wenwen Yan

**Affiliations:** ^1^Department of Critical Care Medicine, Baoding First Central Hospital, Baoding, China; ^2^Department of Research and Development, United Imaging Intelligence (Beijing) Co., Ltd., Beijing, China

**Keywords:** patient outcome assessment, *Mycoplasma pneumonia*, Radiomics, X-ray computed tomography, machine learning

## Abstract

**Objective:**

To develop and validate model based on clinical, imaging, and Radiomics features for predicting disease severity and delayed recovery in *Mycoplasma pneumoniae* pneumonia (MPP).

**Methods:**

This multicenter retrospective study enrolled 238 patients (training cohort), 60 (testing cohort), and 278 (validation cohort). Patients were classified into non-severe MPP (NSMPP) and severe MPP (SMPP) groups based on guideline, and further stratified post-treatment into recovery or delayed recovery groups. Radiomics features were extracted from chest CT using PyRadiomics, with Least Absolute Shrinkage and Selection Operator (LASSO) regression for feature selection. Three random forest-based predictive models were developed, including Clinical-Image, Radiomics, and Integrated. Predictive performance was evaluated via by the area under the receiver operating characteristic curve (AUC), calibration, and clinical utility.

**Results:**

The Integrated model demonstrated superior discrimination for severity prediction (validation AUC: 0.784, 95% CI: 0.722–0.845) and delayed recovery (validation AUC: 0.865, 95% CI: 0.770–0.960), outperforming Clinical-Image (severity AUC: 0.771, 95% CI: 0.695–0.847; delayed recovery AUC: 0.807, 95% CI: 0.724–0.950) and Radiomics model (severity AUC: 0.710, 95% CI: 0.643–0.776; delayed recovery AUC: 0.837, 95% CI: 0.724–0.950). Integrated Discrimination Improvement (IDI) analysis demonstrated significant enhancements in the Integrated model compared to both the Clinical-Image and Radiomics models for predicting both disease severity and delayed recovery (all *p* < 0.05). Key predictors comprised D-dimer (severity OR = 1.371; delayed recovery OR = 4.061), systemic immune-inflammation index (delayed recovery OR = 6.607), and consolidation patterns (delayed recovery OR = 2.820).

**Conclusion:**

The Integrated model combining clinical, imaging, and Radiomics features enhances risk stratification for MPP severity and delayed recovery.

## Introduction

*Mycoplasma pneumoniae* (MP) is a significant cause of respiratory infections in both children and adults, accounting for 10–40% of community-acquired pneumonia cases develop into *Mycoplasma pneumoniae* pneumonia (MPP) (1, 2). School-aged children are most frequently affected, but adults and the elderly are also susceptible (3). In recent years, the global incidence of MPP and severe *Mycoplasma pneumoniae* pneumonia (SMPP) has increased, often leading to complications such as pleural effusion, atelectasis, and even life-threatening extrapulmonary conditions (4, 5). SMPP can involve multiple organ systems and result in long-term sequelae (6, 7), posing major challenges for clinical management and increasing the healthcare burden (8, 9).

SMPP may develop diffuse alveolar hemorrhage, pulmonary embolism, or acute respiratory distress syndrome, resulting in reduced survival rates and long-term morbidity (10, 11). However, current pathogen detection methods suffer from long turnaround times and limited reliability due to false positives or negatives (12–14). Moreover, conventional clinical indicators such as interleukin-6 levels are difficult to obtain rapidly, leaving clinicians with limited tools for early risk stratification. Therefore, timely identification of patients at risk for severe disease and delayed radiological recovery is crucial to improving outcomes and guiding therapy.

Computed tomography (CT) plays a central role in diagnosing MPP, typically typically manifesting as ground-glass opacities, lobar consolidations with air bronchograms, and interlobular septal thickening (15). Yet conventional CT assessments lack reproducibility and are highly operator dependent. Radiomics—an advanced computational technique extracting high-dimensional quantitative features from medical images—has emerged as a powerful tool for uncovering imaging biomarkers not visible to the human eye (16–18). It allows quantitative analysis of shape, texture, and intensity, enabling objective evaluation of disease burden (16). Radiomics has shown promise in assessing disease severity in conditions like COVID-19 (19) and may offer similar benefits in MPP, particularly for distinguishing SMPP and predicting delayed radiographic resolution (20).

This study aims to develop and validate CT-based Radiomics model to stratify SMPP risk and predict delayed recovery. By integrating Radiomics with clinical and conventional imaging data, we seek to establish robust prediction models to support early risk identification and individualized clinical decision-making.

## Materials and methods

### Patient population

This study follows the Declaration of Helsinki and was approved by the Ethics Committee of the Baoding First Central Hospital (grant no. HDFYLL-KY-2024-002) and also waived informed consent. A retrospective collection of cases was conducted at a single medical center between July 2024 and March 2025. These cases were randomly assigned to a training cohort and a testing cohort in an 8:2 ratio. Concurrently, cases from the same period were obtained from two additional medical centers to constitute an external validation cohort.

Inclusion criteria were as follows: (1) patients diagnosed with MPP, regardless of age; and (2) availability of chest CT images and corresponding clinical data. Exclusion criteria included: (1) presence of immunodeficiency disorders, chronic pulmonary diseases, cardiac conditions, chronic glomerulonephritis, rheumatic diseases, malnutrition, diabetes, or other genetic/metabolic disorders; (2) co-infection with other respiratory pathogens; (3) incomplete clinical data; and (4) prior pulmonary surgery.

### Patient grouping

Based on clinical severity at presentation, patients were classified into two groups: NSMPP and SMPP, following national diagnostic criteria (21). NSMPP was defined by typical symptoms of community-acquired pneumonia (CAP)—fever, cough, and abnormal lung auscultation—along with laboratory confirmation of MP infection via elevated MP-specific IgM titers (≥1:160 or a fourfold rise over two weeks) or positive MP specific polymerase chain reaction from nasopharyngeal secretions.

SMPP was diagnosed when patients met any of the severity criteria (21), including persistent high fever (≥39 °C for ≥5 days or ≥7 days without improvement), respiratory distress (e.g., dyspnea, wheezing, hemoptysis), or complications such as plastic bronchitis, pleural effusion, asthma exacerbation, or extrapulmonary involvement. Additional criteria included oxygen saturation ≤93% on room air, or radiographic evidence of extensive lung involvement—e.g., consolidation in ≥ two-thirds of a lobe, high-density lesions in ≥ two lobes, or diffuse bilateral infiltrates. Rapid radiologic progression (>50% within 24–48 h) or significantly elevated inflammatory markers such as C-reactive protein (CRP), lactate dehydrogenase (LDH), or D-dimer, were also supported SMPP classification.

Treatment was severity-based: NSMPP patients received symptomatic care and anti-MP therapy, while SMPP cases received comprehensive management, including broad-spectrum antibiotics, corticosteroids, bronchoscopy, and anticoagulation when indicated.

Recovery outcomes were assessed at 14 days post-treatment initiation, a timepoint supported by prior studies indicating that most non-severe MPP cases achieve clinical and radiological resolution within 10–14 days of appropriate therapy (22). Persistent symptoms or imaging abnormalities beyond this window may indicate treatment resistance, delayed immune recovery, or early fibrosis (23).

**Figure 1:**
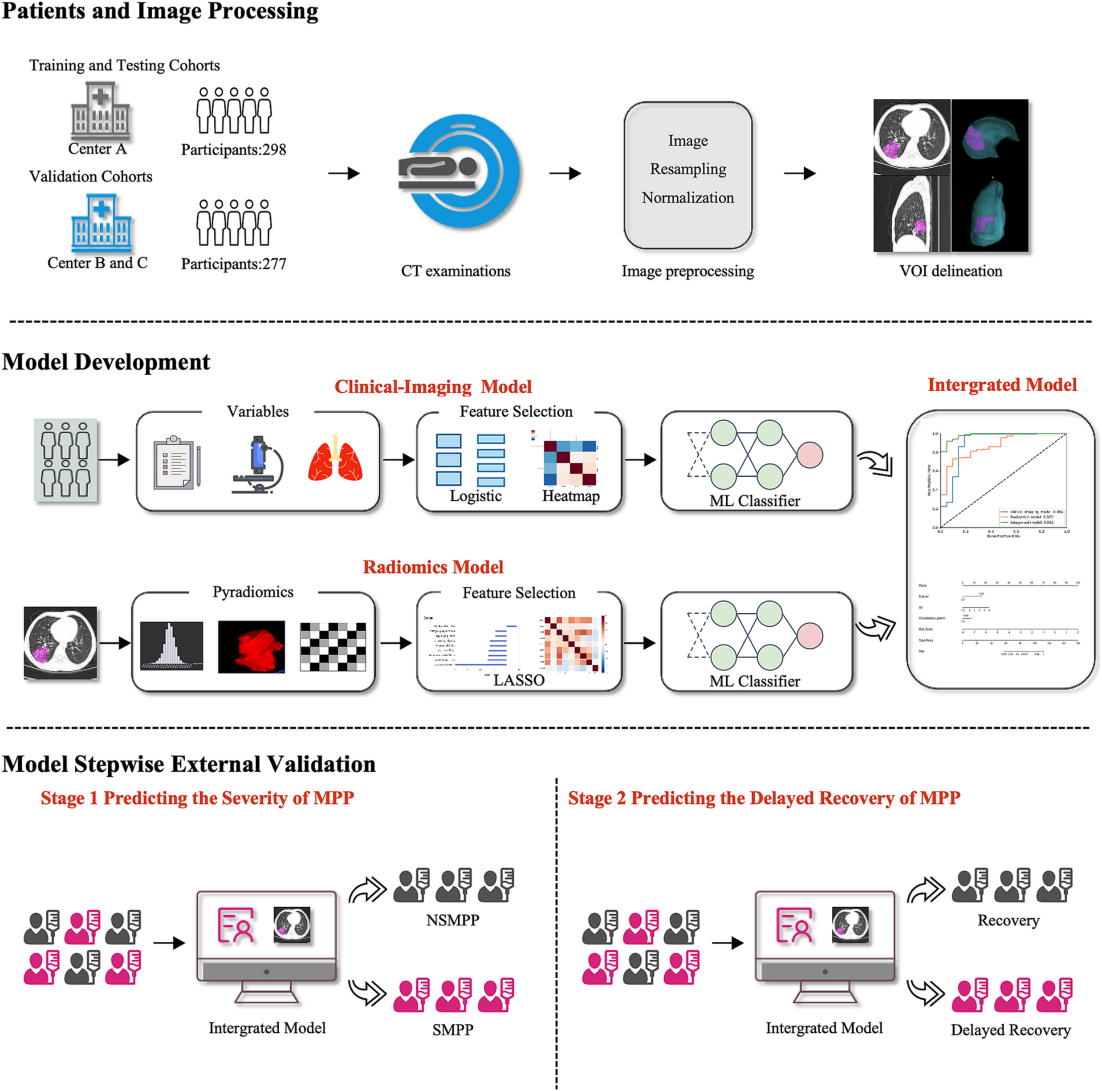
Flow chart of the study. CT, Computed Tomography; VOI, Volume of Interest; ML, Machine Learning; LASSO, Least Absolute Shrinkage and Selection Operator; MPP, *Mycoplasma pneumoniae* Pneumonia; NSMPP, Non-Severe *Mycoplasma pneumoniae* Pneumonia; SMPP, Severe *Mycoplasma pneumoniae* Pneumonia.

Based on this, patients were stratified into recovery or delayed recovery groups. Recovery was defined as the complete resolution of respiratory symptoms and normalization of follow-up chest CT. Delayed recovery was defined by the persistence of any clinical symptoms (e.g., cough, dyspnea, chest discomfort) and/or radiographic abnormalities, including: (1) persistent lobar consolidation, atelectasis, or pleural effusion with no significant resolution compared to baseline; and/or (2) residual lung involvement exceeding 30% of the initially affected lung fields. All follow-up CT images were independently reviewed by two radiologists blinded to clinical outcomes, with disagreements resolved via consensus with a senior thoracic radiologist. A schematic overview is shown in Figure 1.

### Clinical data collection and imaging analysis

Patient data were retrospectively collected and included demographic information, clinical presentation, laboratory findings, and imaging characteristics. General demographic variables encompassed sex, age, and fever classification (low-grade, moderate, or high-grade). Laboratory parameters included white blood cell (WBC) count, fibrinogen (FIB), D-dimer, and the systemic immune-inflammation index (SII). SII was calculated using the following formula: platelet count × neutrophil count/lymphocyte count.

All patients underwent chest CT examinations during their illness. Two radiologists (Radiologist A with 6 years and Radiologist B with 8 years of diagnostic experience) independently reviewed and interpreted the CT images. In cases of disagreement regarding image interpretation, a third senior radiologist (Radiologist C, with 12 years of experience) was consulted to resolve discrepancies and reach a consensus. Throughout the image evaluation process, all radiologists were blinded to clinical data and patient identifiers to minimize bias. The radiological assessment focused on three specific CT features: lobar atelectasis (classified as absent or present), consolidation pattern (categorized as absent, patchy, segmental, or wedge-shaped), and pleural effusion (classified as absent or present).

### Image acquisition and preprocessing

Chest CT scans were performed using various CT scanners, with details of the scanner specifications and scanning parameters provided in Supplementary information and summarized in Supplementary Table S1. All examinations were conducted with patients in the supine position during breath-hold following deep inspiration. Prior to scanning, standardized breath-holding training was provided to ensure image quality. The scan range extended from the thoracic inlet to the costophrenic angles.

Image preprocessing involved resampling to isotropic voxels of 1 mm3 using B-spline interpolation and histogram standardization of intensity values in the CT scans.

### Radiomics feature extraction and selection

Image segmentation, Radiomics feature extraction, feature selection, and machine learning model development were conducted using the uAI Research Portal V1.1 (Shanghai United Imaging Intelligence, Co., Ltd.) (24). An automated segmentation algorithm (25), was employed to delineate the lesion volumes within each pulmonary lobe. The initial automated segmentation outputs underwent rigorous refinement through independent manual annotation by two board-certified radiologists (Radiologist A: 6 years of thoracic imaging experience; Radiologist B: 8 years). To quantify interobserver agreement, intraclass correlation coefficients (ICC, two-way random-effects model for absolute agreement) were calculated across all segmented lesions, with ICC values interpreted as: <0.40 poor; 0.40–0.59 fair; 0.60–0.74 good; >0.75 excellent. Discrepancies exceeding 5% volumetric difference were resolved through consensus review with a senior radiologist (15 years’ experience).

Radiomics feature extraction was performed using the PyRadiomics V3.0 (26) toolkit integrated within the uAI Research Portal V1.1. A total of 1,904 features were automatically extracted from both the original and filtered CT images by applying 15 image filters. These features comprised seven major categories: first-order statistical features (*n* = 378), shape-based (morphological) features (*n* = 14), gray-level co-occurrence matrix (GLCM) features (*n* = 441), gray-level run length matrix (GLRLM) features (*n* = 336), gray-level size zone matrix (GLSZM) features (*n* = 336), gray-level dependence matrix (GLDM) features (*n* = 294), and neighboring gray-tone difference matrix (NGTDM) features (*n* = 105). A detailed description of the applied filters and corresponding feature sets is provided in Supplementary information 2.

After applying Z-score normalization and Spearman correlation analysis, the least absolute shrinkage and selection operator (LASSO) regression addressed feature collinearity.

### Model development

Univariable logistic regression was performed to evaluate the association between clinical and imaging variables with MPP severity and recovery status. Variables with a *p*-value < 0.05 were considered statistically significant and subsequently included in model construction. For Radiomics features, Z-score normalization followed by LASSO regression was applied for feature selection, with features yielding *p*-values < 0.05 retained for model development.

Three random forest models were developed: A Clinical-Image Model incorporating clinical and imaging variables, a Radiomics Model based solely on selected Radiomics features, and an Integrated Model combining clinical, imaging, and Radiomics features.

### Statistical analysis

Continuous variables were reported as median (IQR), while categorical variables were expressed as frequencies and percentages. Comparisons of patient characteristics across the training, testing, and validation cohorts were conducted using the Fisher’s exact test, Pearson’s χ2 test, or the Mann–Whitney U test, as appropriate based on variable type and distribution. Univariable and multivariable logistic regression analyses were also performed to identify potential differences among cohorts.

The predictive performance of the models in assessing the severity and delayed recovery of MPP was evaluated using the Receiver Operating Characteristic curves (ROC) and area under the ROC (AUC) with corresponding 95% confidence intervals. Additional performance metrics including sensitivity, specificity, accuracy, F1 score and precision were calculated to provide a comprehensive assessment of model effectiveness. Pairwise comparisons of model performance were conducted using the DeLong test and integrated discrimination improvement (IDI) analysis. To enhance interpretability of the Integrated models, we performed SHAP (SHapley Additive Explanations) analysis to evaluate the relative contribution of each feature to the model’s output.

All statistical analyses were performed using R software (version 3.6.0; The R Foundation for Statistical Computing). A two-sided *p*-value < 0.05 was considered statistically significant.

## Results

### Patient characteristics

**Figure 2:**
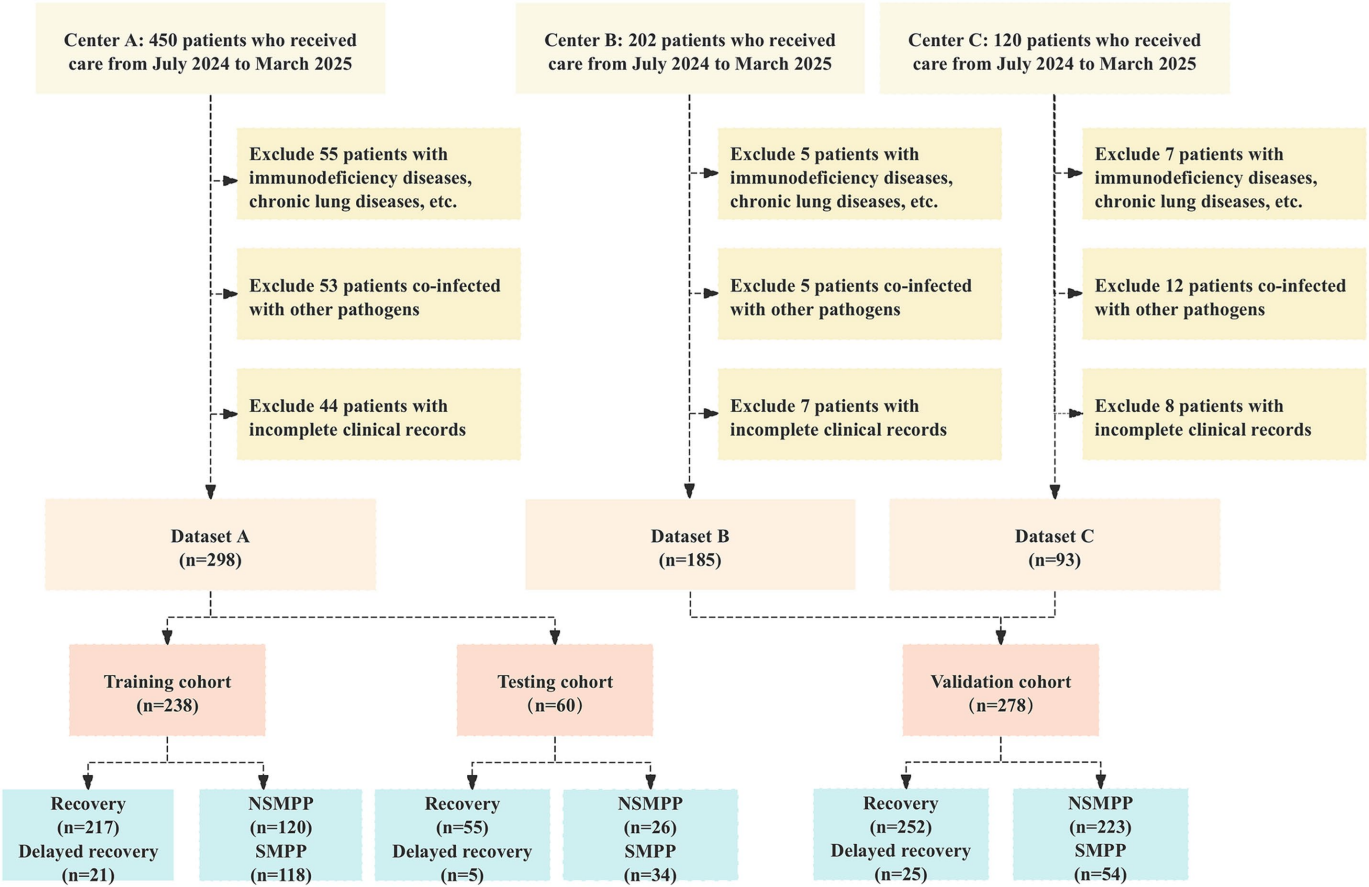
Flowchart of participant selection: adherence to inclusion and exclusion criteria in a multicenter cohort study. NSMPP, Non-Severe *Mycoplasma pneumoniae* Pneumonia; SMPP, Severe *Mycoplasma pneumoniae* Pneumonia.

A total of 238 patients were included in the training cohort, while 60 patients comprised the testing cohort. Additionally, 278 patients from external centers were enrolled in the validation cohort. The detailed inclusion and exclusion process is depicted in Figure 2.

Significant inter-cohort differences (all *P*-inter <0.05) were observed in age, type of fever, fibrinogen (FIB), D-dimer, systemic immune inflammation index (SII), lobar atelectasis, consolidation pattern, and pleural effusion between NSMPP and SMPP groups (Table 1). Marked inter-cohort differences (all *P*-inter <0.05) were observed in age, type of fever, FIB, SII, lobar atelectasis, consolidation pattern, and pleural effusion between recovery and delayed recovery groups (Table 2).

**Table 1:** Comparison of clinical and radiological characteristics between NSMPP and SMPP groups in training, testing and validation cohorts.

Characteristics	Training cohort (*n* = 238)			Testing cohort (*n* = 60)			Validation cohort (*n* = 277)			Overall (*n* = 575)
	NSMPP (*n* = 120)	SMPP (*n* = 118)	*P*-Intra value	NSMPP (*n* = 26)	SMPP (*n* = 34)	*P*-Intra value	NSMPP (*n* = 223)	SMPP (*n* = 54)	*P* intra value	*P*-inter value
Male, *n* (%)	59 (49.167)	69 (58.475)	0.150	9 (34.615)	23 (67.647)	0.011	101 (45.291)	24 (44.444)	0.911	0.121
Age, M (IQR), years	34.000 [10.000,65.000]	40.000 [6.250,68.750]	0.747	47.000 [9.500,57.000]	33.000 [8.000,66.000]	0.988	33.000 [8.000,66.000]	48.000 [10.000,58.000]	0.082	<0.001
Type of fever, *n* (%)			0.061			0.629			<0.001	<0.001
Absent	58 (48.333)	44 (37.288)		24 (40.000)	13 (50.000)		23 (10.314)	2 (3.704)		
Low-grade	7 (5.833)	17 (14.407)		5 (8.333)	2 (7.692)		23 (10.314)	1 (1.852)		
Mid-grade	26 (21.667)	21 (17.797)		14 (23.333)	6 (23.077)		104 (46.637)	17 (31.481)		
Hyperpyrexia	29 (24.167)	36 (30.508)		17 (28.334)	5 (19.231)		73 (32.735)	34 (62.963)		
WBC, M (IQR), 109/L	6.555 [4.683,8.807]	8.035 [5.955,10.555]	0.001	7.455 [6.357,9.300]	9.265 [6.747,11.890]	0.182	7.270 [5.930,9.290]	9.460 [6.710,11.630]	0.001	0.068
FIB, M (IQR), 109/L	3.845 [3.080,4.615]	4.190 [3.655,4.850]	0.017	3.705 [3.093,4.333]	4.485 [3.660,5.692]	0.015	3.670 [3.010,4.235]	3.785 [3.370,4.357]	0.144	<0.001
D-dimer, *n* (%)			0.003			0.916			0.049	<0.001
0–500 ug/L	34 (14.286)	15 (12.712)		15(57.692)	18(52.941)		40 (17.937)	10 (18.519)		
>500 ug/L	204 (85.714)	103 (87.288)		11(42.308)	16(47.059)		183 (82.063)	44 (81.481)		
SII, M (IQR)	567.798 [355.964,917.839]	871.539 [523.636,1672.732]	<0.001	820.135 [470.647,1284.275]	1011.183 [508.730,2182.868]	0.230	582.950 [374.220,895.845]	883.830 [595.239,1569.793]	<0.001	0.004
Lobar atelectasis			0.819			1.000			0.127	0.001
Absent	99 (82.500)	96 (81.356)		25 (96.154)	33 (97.059)		205 (91.928)	46 (85.185)		
Present	21 (17.500)	22 (18.644)		1 (3.846)	1 (2.941)		18 (8.072)	8 (14.815)		
Consolidation pattern			0.104			0.526			<0.001	0.025
Absent	21 (17.500)	31 (26.271)		3 (11.538)	5 (14.706)		46 (20.628)	4 (7.407)		
Patchy	38 (31.667)	28 (23.729)		2 (7.692)	5 (14.706)		69 (30.942)	11 (20.370)		
Segmental	26 (21.667)	34 (28.814)		14 (53.846)	12 (35.294)		67 (30.045)	14 (25.926)		
Wedge-shaped	35 (29.167)	25 (21.186)		7 (26.923)	12 (35.294)		41 (18.386)	25 (46.296)		
Pleural effusion			0.638			0.597			0.003	<0.001
Absent	85 (70.833)	89 (75.424)		24 (92.308)	30 (88.235)		213 (95.516)	45 (83.333)		
Present	35 (29.167)	29 (24.576)		2 (7.692)	4 (11.765)		10 (4.485)	9 (16.667)		

*P*-intra value is the result of univariate analyses between the NSMPP and SMPP groups; *P*-inter value represents the comparisons of characteristics between training testing and validation cohorts. NSMPP, Non Severe *Mycoplasma pneumoniae* Pneumonia; SMPP, Severe *Mycoplasma pneumoniae* Pneumonia; Interquartile range (IQR); WBC, white blood cell; FIB, Fibrinogen; SII, Systemic immune inflammation index.

**Table 2:** Comparison of clinical and radiological characteristics between recovery and delayed recovery groups in training, testing and validation cohorts.

Characteristics	Training cohort (*n* = 238)			Testing cohort (*n* = 60)			Validation cohort (*n* = 277)			Overall (*n* = 575)
	Recovery (*n* = 217)	Delayed recovery (*n* = 21)	P-intra value	Recovery (*n* = 55)	Delayed recovery (n = 5)	P-intra value	Recovery (*n* = 252)	Delayed recovery (*n* = 25)	P-intra value	P-inter value
Male, *n* (%)	112 (51.613)	13 (61.905)	0.367	28 (50.909)	2 (40.000)	1.000	118 (46.825)	12 (48.000)	0.911	0.448
Age, M (IQR), years	38.000 [8.000,66.000]	52.000 [8.000,68.000]	0.910	30.000 [9.000,69.500]	13.000 [3.000,57.000]	0.297	26.000 [6.000,46.250]	32.000 [12.000,48.000]	0.282	<0.001
Type of fever, *n* (%)			0.410			0.826			0.081	<0.001
Absent	106 (48.848)	11 (52.381)		24 (43.636)	2 (40.000)		21 (8.333)	4 (16.000)		
Low-grade	22 (10.138)	4 (19.048)		6 (10.909)	0 (0.000)		19 (7.540)	5 (20.000)		
Mid-grade	41 (18.894)	4 (19.048)		11 (20.000)	2 (40.000)		113 (44.841)	8 (32.000)		
Hyperpyrexia	48 (22.120)	2 (9.524)		14 (25.455)	1 (20.000)		99 (39.286)	8 (32.000)		
WBC, M (IQR), 109/L	7.500 [5.790,10.130]	5.560 [4.360,8.120]	0.044	6.800 [5.035,9.400]	4.490 [4.280,13.930]	0.593	7.475 [6.018,9.752]	8.260 [6.470,10.330]	0.232	0.591
FIB, M (IQR), 109/L	4.080 [3.310,4.890]	3.760 [3.030,4.480]	0.079	3.930 [3.470,4.675]	4.760 [4.100,7.240]	0.204	3.370 [0.548,3.960]	3.620 [0.550,4.360]	0.153	<0.001
D-dimer, *n* (%)			<0.001			0.015			<0.001	0.208
0–500 ug/L	20 (9.217)	17 (80.952)		11 (20.000)	4 (80.000)		36 (14.286)	17 (68.000)		
>500 ug/L	197 (90.783)	4 (19.048)		44 (80.000)	1 (20.000)		216 (85.714)	8 (32.000)		
SII, M (IQR)	521.150 [251.830,875.400]	181.000 [127.000,243.000]	<0.001	286.000 [80.000,635.350]	406.590 [201.520,426.040]	0.487	408.146 [381.993,511.541]	619.183 [374.268,828.072]	0.003	0.001
Lobar atelectasis			<0.001			0.308			0.799	<0.001
Absent	46 (21.198)	11 (52.381)		40 (72.727)	2 (40.000)		166 (65.873)	17 (68.000)		
Prsent	171 (78.802)	10 (47.619)		15 (27.273)	3 (60.000)		86 (34.127)	8 (32.000)		
Consolidation pattern			<0.001			0.008			<0.001	<0.001
Absent	29 (13.364)	8 (38.095)		10 (18.182)	4 (80.000)		21 (8.333)	11 (44.000)		
Patchy	39 (17.972)	10 (47.619)		4 (7.273)	1 (20.000)		89 (35.317)	11 (44.000)		
Segmental	112 (51.613)	3 (14.286)		36 (65.455)	0 (0.000)		82 (32.540)	2 (8.000)		
Wedge-shaped	37 (17.051)	0 (0.000)		5 (9.091)	0 (0.000)		60 (23.810)	1 (4.000)		
Pleural effusion			0.813			1.000				<0.001
Absent	167 (76.959)	17 (80.952)		40 (72.727)	4 (80.000)		233 (92.460)	25 (100.000)		
Prsent	50 (23.041)	4 (19.048)		15 (27.273)	1 (20.000)		19 (7.540)	0 (0.000)		

*P*-intra is the result of univariate analyses between the recovery and delayed recovery groups; *P*-inter value represents the comparisons of characteristics between training testing and validation cohorts. NSMPP, Non Severe *Mycoplasma pneumoniae* Pneumonia; SMPP, Severe *Mycoplasma pneumoniae* Pneumonia; Interquartile range (IQR); WBC, white blood cell; FIB, Fibrinogen; SII, Systemic immune inflammation index.

### Variables associated with MPP severity and delayed recovery in the training dataset

For severity stratification, white blood cell (WBC; OR = 1.672, *p* = 0.001), D-dimer (OR = 1.659, *p* = 0.007), and SII (OR = 2.125, *p* < 0.001) showed significant associations. Multivariate analysis retained only D-dimer as an independent predictor (OR = 1.371, *p* = 0.050).

In contrast, delayed recovery demonstrated stronger associations with D-dimer (OR = 3.869, *p* < 0.001), SII (OR = 4.734, *p* = 0.001), fibrinogen (FIB, OR = 2.024, *p* = 0.047), lobar atelectasis (OR = 1.676, *p* = 0.010), and consolidation pattern (OR = 2.850, *p* < 0.001) in univariate analysis. The multivariate model identified three robust predictors: D-dimer (OR = 4.061, *p* < 0.001), SII (OR = 6.607, *p* = 0.001), and consolidation pattern (OR = 2.820, *p* = 0.001) (Table 3).

**Table 3:** Univariate and multivariable logistic regression analyses for selecting clinical and radiological features in the training cohort.

Predictor	Univariate logistic regression			Multivariate logistic regression		
	Coefficient	OR (95% CI)	*P*-value	coefficient	OR (95% CI)	*P*-value
Severity						
Male	0.007	1.007 (0.781, 1.299)	0.955			
Age	0.030	1.030 (0.799, 1.328)	0.819			
Type of fever	0.156	1.169 (0.906, 1.509)	0.229			
WBC	0.514	1.672 (1.240, 2.253)	0.001			
FIB	0.260	1.297 (0.989, 1.700)	0.060			
D-dimer	0.506	1.659 (1.147, 2.400)	0.007	0.316	1.371 (1.001, 1.879)	0.050
SII	0.754	2.125 (1.478, 3.057)	<0.001			
Lobar atelectasis	0.030	1.030 (0.799, 1.328)	0.819			
Consolidation pattern	−0.162	0.851 (0.659, 1.098)	0.215			
Pleural effusion	−0.058	0.944 (0.731, 1.218)	0.655			
Delayed recovery						
Male	0.211	1.230 (0.786, 1.953)	0.370			
Age	−0.013	0.991 (0.632, 1.554)	0.965			
Type of fever	0.247	1.280 (0.793, 2.066)	0.312			
WBC	0.234	1.263 (0.754, 2.114)	0.374			
FIB	0.705	2.024 (1.011, 4.055)	0.047			
D-dimer	1.353	3.869 (2.521, 5.939)	<0.001	1.401	4.061 (2.518, 6.547)	<0.001
SII	1.555	4.734 (1.909, 11.740)	0.001	1.888	6.607 (2.078, 21.005)	0.001
Lobar atelectasis	0.517	1.676 (1.129, 2.490)	0.010			
Consolidation pattern	1.047	2.850 (1.740, 4.667)	<0.001	1.037	2.820 (1.517, 5.243)	0.001
Pleural effusion	0.293	1.340 (0.737, 2.440)	0.338			

WBC, white blood cell; FIB, Fibrinogen; SII, Systemic immune inflammation index; OR, odds ratio; CI, Confidence Interval.

### Diagnostic performance of different models for predicting severity in MPP

**Figure 3:**
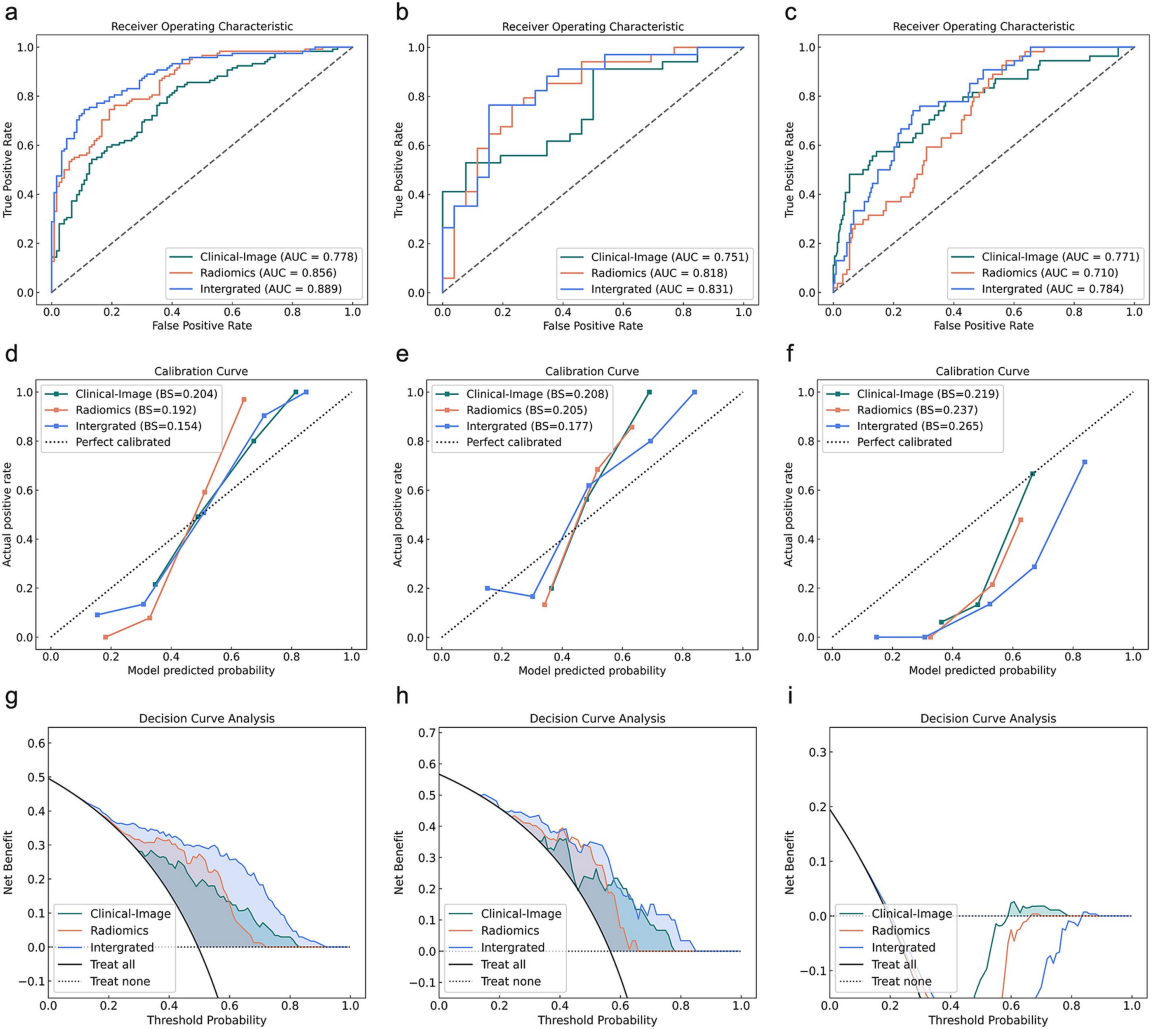
Predictive performance of Clinical-Image, Radiomics, and Integrated models across training, testing, and validation cohorts for severity stratification. **(a–c)** ROC curves, **(d–f)** Calibration curves, **(g–i)** Decision curves for training (left), testing (middle), and validation (right) cohorts. AUC, area under the receiver operating curve.

Figure 3 demonstrates the ROC curves, calibration curves, and decision curves of the three predictive models for severity stratification. For severity prediction, the Clinical-Image model incorporated 1 feature (D-diamer), the Radiomics model included 13 Radiomics features, and the Integrated model combined both (total 14 features). The Clinical-Image model achieved an AUC of 0.771 (95% CI: 0.695–0.847) with 69.0% accuracy in the validation cohort. The Radiomics model showed slightly lower performance (AUC = 0.710, 95% CI: 0.643–0.776; accuracy = 52.7%). In the validation cohort, the Clinical-Image model (incorporating 1 clinical feature) achieved an AUC of 0.771 (95% CI: 0.695–0.847). The Radiomics model (13 Radiomics features) demonstrated an AUC of 0.710 (95% CI: 0.643–0.776). The Integrated model (combining 14 features) yielded an AUC of 0.784 (95% CI: 0.722–0.845) (Table 4).

**Table 4:** Diagnostic performance of different models for predicting severity and delayed recovery in MPP.

Model	AUC (95% CI)	Accuracy	F1 Score	Precision	Sensitivity	Specificity
Severity						
Training cohort						
Clinical-image model	0.778 (0.720, 0.836)	0.693	0.640	0.765	0.551	0.833
Radiomics model	0.846 (0.799, 0.894)	0.744	0.745	0.712	0.781	0.710
Integrated model	0.889 (0.848, 0.930)	0.803	0.800	0.803	0.797	0.808
Testing cohort						
Clinical-image model	0.751 (0.629,0.874)	0.667	0.615	0.889	0.471	0.923
Radiomics model	0.818 (0.706, 0.930)	0.767	0.788	0.813	0.765	0.769
Integrated model	0.831 (0.725, 0.938)	0.783	0.794	0.862	0.735	0.846
Validation cohort						
Clinical-image model	0.771 (0.695, 0.847)	0.690	0.463	0.349	0.685	0.691
Radiomics model	0.710 (0.643, 0.776)	0.527	0.438	0.285	0.944	0.426
Integrated model	0.784 (0.722, 0.845)	0.480	0.419	0.268	0.963	0.363
Delayed recovery						
Training cohort						
Clinical-image model	0.894 (0.791, 0.996)	0.832	0.900	0.984	0.829	0.857
Radiomics model	0.872 (0.798, 0.947)	0.807	0.885	0.967	0.816	0.714
Integrated model	0.982 (0.961, 1.000)	0.924	0.957	0.990	0.926	0.905
Testing cohort						
Clinical-image model	0.865 (0.737, 0.994)	0.733	0.833	0.976	0.727	0.800
Radiomics model	0.865 (0.707, 1.000)	0.767	0.860	0.956	0.782	0.600
Integrated model	0.978 (0.939, 1.000)	0.917	0.953	0.981	0.927	0.800
Validation cohort						
Clinical-image model	0.807 (0.724, 0.950)	0.841	0.908	0.964	0.857	0.680
Radiomics model	0.837 (0.724, 0.950)	0.877	0.930	0.970	0.893	0.720
Integrated model	0.865 (0.770, 0.960)	0.913	0.951	0.971	0.933	0.720

MPP, *Mycoplasma pneumoniae* Pneumonia; AUC, area under the receiver operating characteristic curve; CI, Confidence Interval.

In the validation cohort, the Integrated Discrimination Improvement (IDI) analysis demonstrated significant improvements for the Integrated model compared to the Clinical-Image model (*p* = 0.010) and the Radiomics model (*p* < 0.001). However, the Delong test for AUC differences found no statistically significant superiority of the Integrated model over the Clinical-Image model (*p* = 0.765) or the Radiomics model (*p* = 0.072). Direct comparisons between the Clinical-Image and Radiomics models revealed no significant differences in either AUC (*p* = 0.235) or IDI (*p* = 0.100) (Table 5).

**Table 5:** Comparison of different models in predicting severity and delayed recovery in MPP.

Method	Delong test		IDI	
	Z value	*p* value	Z value	*p* value
Severity				
Training cohort				
Clinical-image vs. Radiomics	2.261	0.024	0.952	0.341
Clinical-image vs. Intergrated	4.236	<0.001	9.326	<0.001
Radiomics vs. Intergrated	1.719	0.086	8.629	<0.001
Testing cohort				
Clinical-image vs. Radiomics	0.838	0.402	0.079	0.937
Clinical-image vs. Intergrated	1.412	0.158	3.110	0.002
Radiomics vs. Intergrated	0.256	0.798	3.408	0.001
Validation cohort				
Clinical-image vs. Radiomics	1.189	0.235	−1.646	0.100
Clinical-image vs. Intergrated	0.299	0.765	2.566	0.010
Radiomics vs. Intergrated	1.798	0.072	5.192	<0.001
Delayed recovery				
Training cohort				
Clinical-image vs. Radiomics	0.311	0.756	−1.522	0.128
Clinical-image vs. Intergrated	1.782	0.075	3.110	0.002
Radiomics vs. Intergrated	3.185	0.001	4.923	0.000
Testing cohort				
Clinical-image vs. Radiomics	0.000	1.000	1.565	0.118
Clinical-image vs. Intergrated	1.952	0.051	3.677	<0.001
Radiomics vs. Intergrated	1.673	0.094	2.305	0.021
Validation cohort				
Clinical-image vs. Radiomics	0.471	0.638	−1.216	0.224
Clinical-image vs. Intergrated	1.947	0.052	4.327	<0.001
Radiomics vs. Intergrated	0.644	0.520	4.316	<0.001

MPP, *Mycoplasma pneumoniae* Pneumonia; IDI, Integrated Discrimination Improvement.

### Diagnostic performance of different models for delayed recovery prediction in MPP

**Figure 4:**
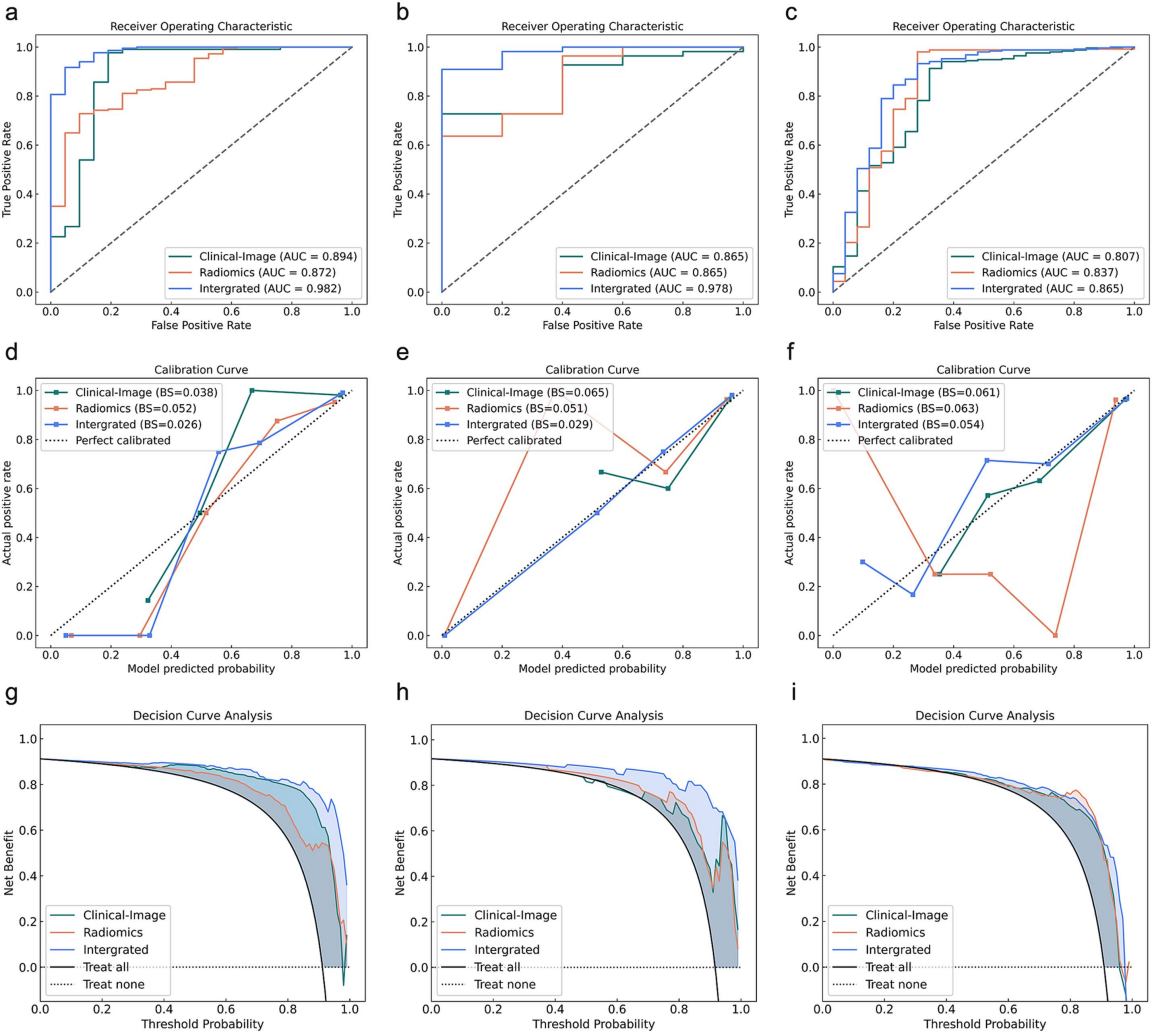
Comparative analysis of Clinical-Image, Radiomics, and Integrated models across training, testing, and validation cohorts for delayed recovery. **(a–c)** ROC curves, **(d–f)** Calibration curves, **(g–i)** Decision curves for training (left), testing (middle), and validation (right) cohorts. AUC, area under the receiver operating curve.

Figure 4 displays the comparative performance of three delayed recovery prediction models through ROC analysis, calibration plots, and decision curve evaluation. The Clinical-Image model, incorporating D-dimer, SII, and consolidation pattern, achieved an AUC of 0.807 (95% CI: 0.724–0.950) with 84.1% accuracy, 85.7% sensitivity, and 68.0% specificity in the validation cohort. The Radiomics model (13 Radiomics features) demonstrated an AUC of 0.837 (95% CI: 0.724–0.950), an accuracy of 87.7%, sensitivity of 89.3%, and specificity of 72.0%. The Integrated model (combining 16 features) yielded an AUC of 0.865 (95% CI: 0.770–0.960), with an accuracy of 91.3%, sensitivity of 93.3%, and specificity of 72.0% (Table 4).

In the validation cohort, while IDI analysis showed the Integrated model’s significant improvement over both Clinical-Image (*p* < 0.001) and Radiomics models (*p* < 0.001), Delong tests revealed non-significant advantage over Clinical-Image (*p* = 0.052) and Radiomics (*p* = 0.520). Direct comparisons between Clinical-Image and Radiomics models showed no significant differences in either AUC (*p* = 0.638) or IDI (*p* = 0.224) (Table 5).

**Figure 5:**
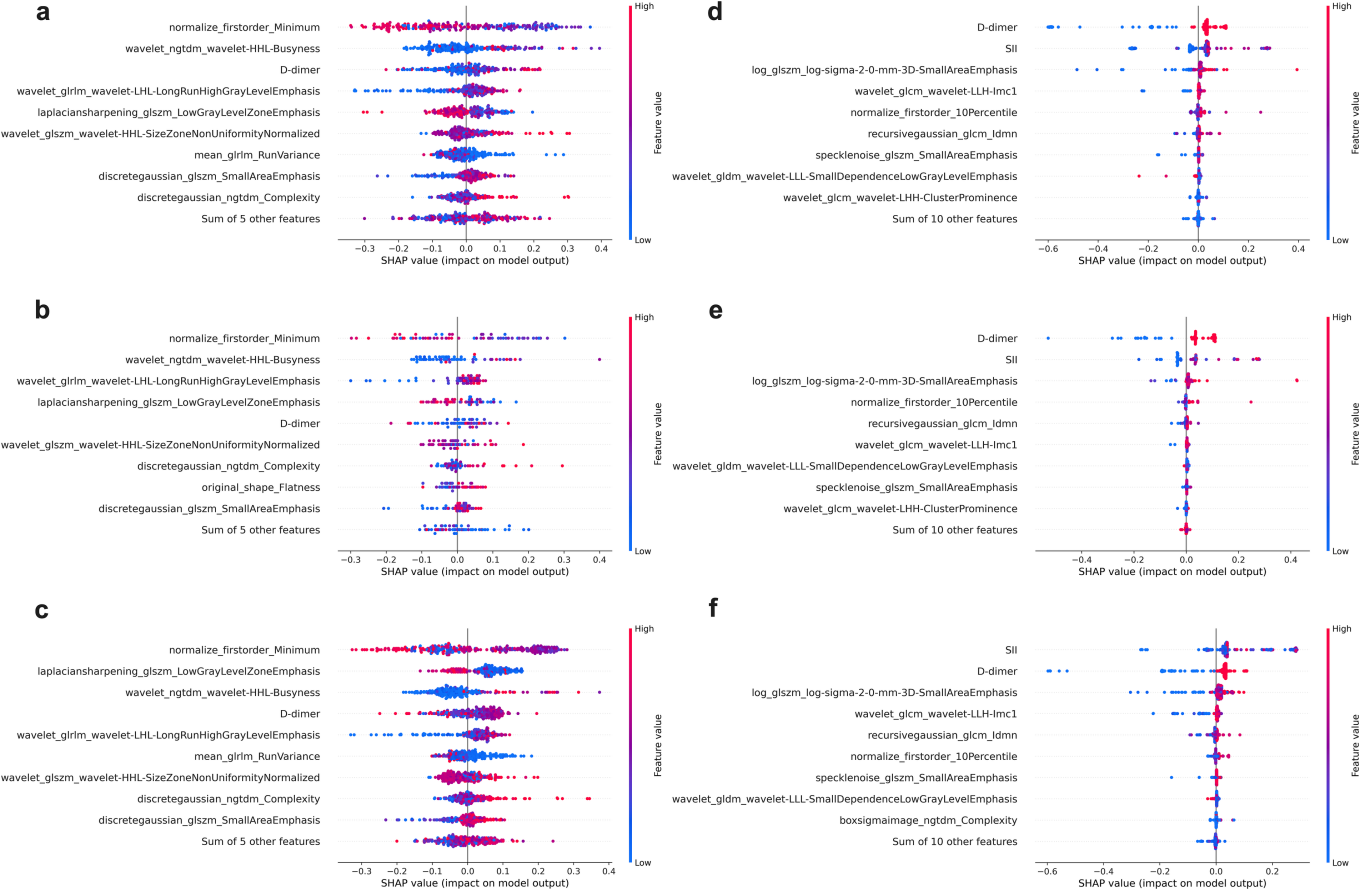
SHAP beeswarm plots for feature contribution analysis in the Integrated models. **(a–c)** Display the top contributing features for the severity prediction model across the training **(a)**, testing **(b)**, and validation **(c)** cohorts, while **(d–f)** show the corresponding SHAP results for the delayed recovery prediction model in the training **(d)**, testing **(e)**, and validation **(f)** cohorts. Each point represents an individual patient, with color indicating the feature value (red = high, blue = low).

As shown in Figure 5, for the severity prediction model (panels a–c), the most influential features included clinical variables (D-dimer), alongside radiomics features in validation cohorts. Similarly, for the delayed recovery prediction model (panels d–f), top contributors included D-dimer, SII, consolidation pattern and radiomic texture features. Notably, clinical biomarkers and radiomics variables appeared synergistic rather than redundant, suggesting that integrating both data types offers complementary predictive value.

## Discussion

This study demonstrates that integrating clinical-radiological features with Radiomics signatures significantly improves prediction of both MPP severity and delayed recovery. The combined model showed strong generalizability in external validation, with an AUC of 0.865 (95% CI: 0.770–0.960) for delayed recovery prediction, underscoring its potential to guide risk-adapted interventions.

Elevated D-dimer levels were identified as an independent predictor of MPP severity (*p* = 0.050), reflecting its pivotal role in hypercoagulability and endothelial dysfunction during disease progression (27). In SMPP, systemic inflammation triggers a prothrombotic state through cytokine-mediated activation of the coagulation cascade, leading to fibrin deposition and microvascular thrombosis (28). D-dimer, as a degradation product of cross-linked fibrin, serves as a biomarker of this pathological process (29). Our findings align with evidence linking hypercoagulability to alveolar damage and hypoxemia in severe pneumonia (30), suggesting that D-dimer elevation may exacerbate tissue hypoxia by impairing pulmonary perfusion, thereby worsening clinical outcomes.

The strong association of D-dimer (OR = 4.06, *p* < 0.001) and SII (OR = 6.61, *p* = 0.001) with delayed recovery underscores the interplay between coagulation abnormalities and sustained inflammation in prolonging disease resolution. Persistently elevated D-dimer levels likely indicate unresolved microthrombosis and endothelial injury, which impair tissue repair and perpetuate hypoxia-driven damage (28). Concurrently, SII—a composite marker integrating neutrophils, platelets, and lymphocytes—reflects a dysregulated immune response characterized by neutrophil-dominated inflammation and insufficient lymphocyte-mediated resolution (31). Notably, the synergy between D-dimer and SII in the integrated model highlights their complementary roles: while D-dimer marks ongoing vascular injury, SII quantifies the inflammatory burden that sustains tissue damage (32, 33). Our data suggest that high SII values may drive delayed recovery by maintaining a pro-inflammatory milieu that inhibits epithelial regeneration and promotes fibrosis.

Consolidation pattern independently predicted delayed recovery in MPP (*p* = 0.001). Radiologically, consolidation represents dense inflammatory exudates within alveolar spaces, leading to impaired gas exchange, reduced mucociliary clearance, and a local environment that favors secondary infections and hypoxia-induced injury (34). This imaging feature aligns with the established pathophysiological understanding that alveolar inflammation and structural lung damage are key contributors to prolonged disease resolution (20, 35). The coexistence of consolidation with elevated D-dimer and SII in our cohort further underscores this mechanistic triad: vascular injury (reflected by D-dimer), neutrophil-dominated hyperinflammation (SII), and structural lung damage (consolidation) collectively perpetuate tissue hypoxia and repair delays. Clinically, these findings advocate for early stratification of patients with consolidation patterns to guide targeted interventions (36), such as adjunctive corticosteroids to mitigate inflammation or proactive pulmonary rehabilitation to prevent fibrotic sequelae.

Age differed significantly between NSMPP and SMPP groups and between recovery subgroups (*p* < 0.001 in all comparisons). This may reflect demographic bias in external cohorts, as children were more likely to seek care at pediatric hospitals, affecting the age distribution.

The Radiomics model demonstrated robust performance in predicting both severity (AUC = 0.710 in validation cohort) and delayed recovery (AUC = 0.837 in validation cohort), underscoring its unique ability to quantify subvisual heterogeneity in lung lesions that conventional clinical metrics may overlook. Radiomics features, such as texture irregularity, spatial gray-level co-occurrence, and volumetric asymmetry, likely reflect microscale pathological processes including alveolar inflammation, microvascular thrombosis, and early fibrosis that drive disease progression. Prior studies in COVID-19 (37) and bacterial pneumonia (38) have shown that Radiomics signatures correlate with hallmarks of prolonged recovery.

The incorporation of SHAP analysis offers valuable insight into the internal decision-making process of the random forest models, addressing the common concern of “black box” limitations in AI applications. In both severity and recovery prediction models, clinical indicators such as D-dimer, SII, and consolidation pattern emerged as dominant contributors—reinforcing their established prognostic relevance in MPP.

Notably, SHAP analysis also highlighted radiomics features that captured subtle intralesional patterns often invisible to visual assessment. In the severity model (Figure 5c), features such as normalize_first order_Minimum and laplacian sharpening_glszm_Low Gray Level Zone Emphasis were among the most influential. These features reflect texture complexity and intra-lesional heterogeneity (39), consistent with severe parenchymal damage and multi-lobar consolidation.

In the delayed recovery model (Figure 5f), the top-ranked features included log_glszm_log-sigma-2-0-mm-3D-SmallAreaEmphasis wavelet_glcm_wavelet-LLH-Imc1. The former quantifies local texture uniformity after wavelet decomposition, with lower values suggesting heterogeneous tissue repair or uneven lesion resolution, which may delay clinical recovery. The latter measures entropy of intensity differences across pixel pairs, with higher values reflecting spatial irregularity and lingering microstructural disorganization—hallmarks of ongoing inflammation or evolving fibrosis (40). These findings underscore the complementary value of radiomics in quantifying microstructural complexity and disease heterogeneity. When integrated with clinical biomarkers, these features enhance the model’s ability to provide pathophysiologic grounded and interpretable predictions, thereby increasing transparency and clinical trust.

Additionally, we observed that the Radiomics model for severity prediction in the validation cohort demonstrated a moderate AUC (0.710) but relatively low accuracy (52.7%), which may appear discordant. This discrepancy can be attributed to several factors. First, the validation cohort exhibited class imbalance (223 NSMPP vs. 54 SMPP), which can skew accuracy metrics while having less impact on AUC, a threshold-independent measure. Second, the model used a default probability threshold for classification; this may not be optimal in an imbalanced dataset and could lead to suboptimal accuracy despite fair discrimination (41). Third, inter-center variations in CT acquisition protocols and scanner types may have reduced the stability of radiomics features across institutions, compromising model generalizability. Finally, the relatively subtle radiographic manifestations in NSMPP cases may reduce feature contrast, particularly affecting Radiomics-only models. These factors collectively contribute to the observed accuracy-AUC discrepancy, which is commonly reported in radiomics studies under similar constraints (42).

Our findings demonstrate that the Integrated model significantly outperformed single modality approaches in both severity stratification and delayed recovery prediction, as evidenced by its superior IDI in validation cohorts. This may be attributed to the ability of Radiomics to augment clinical data by capturing orthogonal biological information (43). For instance, Radiomics features characterizing the heterogeneity of consolidation pattern may act synergistically with elevated SII to predict delayed recovery, as both are indicative of neutrophil-driven inflammation and ongoing tissue remodeling (44). Additionally, enhancement in discriminative performance of Integrated model underscores the synergistic value of combining pathophysiological biomarkers (D-dimer, SII), quantitative imaging signatures (consolidation patterns), and Radiomics features enabling a more granular risk assessment that aligns with the multifactorial nature of pneumonia progression. Consequently, the Integrated model’s risk assessment capability facilitates decision-making and therapeutic adjustment.

### Limitations

While this study highlights the clinical potential of the Integrated model, several limitations merit consideration. First, the retrospective design may introduce selection bias and overestimate model performance relative to prospective applications. Second, the absence of longitudinal imaging and biomarker data restricts our ability to assess evolving processes such as macrolide resistance or delayed fibrosis. Third, although an external validation cohort was included, all centers shared similar infrastructure, potentially limiting generalizability to low-resource settings. Additionally, the model’s reliance on high-resolution CT and application of pediatric-based severity criteria to adults may reduce applicability and introduce classification bias. Lastly, treatment regimens were heterogeneous and incompletely documented, limiting our ability to control for therapeutic effects on recovery outcomes. Future studies should incorporate standardized imaging protocols, age-adapted severity frameworks, and detailed treatment data to enhance model robustness and clinical relevance.

## Conclusion

This study demonstrates that the Integrated model, combining clinical imaging and Radiomics features, significantly improves predictive accuracy for both MPP severity and delayed recovery. The identification of D-dimer, SII, and consolidation patterns as robust independent predictors underscores their critical roles in disease progression. By stratifying patients into distinct risk categories based on these biomarkers, the model facilitates targeted clinical decision-making and therapeutic adjustments, aligning with precision medicine principles to optimize individualized management strategies.

## Data Availability

The datasets presented in this article are not readily available because the datasets generated during the current study are not publicly available due to hospital regulations and confidentiality agreements, but they are available from the corresponding author upon reasonable request and with necessary ethical approvals. Requests to access the datasets should be directed to liqian202505@163.com.
